# 752 Baking Bread and Other New Hobbies: Characterizing Burn Center Admissions During the COVID-19 Pandemic

**DOI:** 10.1093/jbcr/irac012.305

**Published:** 2022-03-23

**Authors:** Melissa M McLawhorn, Sarah E Burkey, Cara Delatore, Melissa Brantley, Lauren T Moffatt, Laura S Johnson, Jeffrey W Shupp

**Affiliations:** MedStar Health Research Institute, Washington, District of Columbia; MedStar Health Research Institute, Washington, District of Columbia; MedStar Health Research Institute, Washington, District of Columbia; MedStar Washington Hospital Center, Washington, District of Columbia; Burn Center at MedStar Washington Hospital Center, Washington DC, District of Columbia; MedStar Washington Hospital Center / Georgetown School of Medicine, Washington, District of Columbia; MedStar Washington Hospital Center, Washington DC, District of Columbia

## Abstract

**Introduction:**

The effects of the ongoing COVID19 pandemic are wide-reaching and still emerging. Fear of the virus, public health messaging, and government-instituted lockdowns have altered how Americans live, work, and use the healthcare system. There is minimal data that assesses how the COVID-19 pandemic and associated stay at home orders have influenced the etiology of burn injuries. With the majority of burns occurring in the home, it is possible lock down orders have significantly impacted etiology of major burn injuries. This project aims to characterize the demographics and injury characteristics of burn patients seen at a regional burn center during the COVID-19 pandemic.

**Methods:**

Following IRB approval, our institution queried it’s burn registry from March 2020-June 2021. Data on demographics, injury circumstance and details, interventions, COVID-19 status, and outcomes were collected. Descriptive statistics were obtained for the population.

**Results:**

There were 622 inpatient admissions during the study timeframe. Patients were primarily Black (44.4%) or Caucasian (32.6%) males (65.6%) identifying as Non-Hispanic (81.8%). The mean age was 46.73±18.6 years. Mean total TBSA burned was 6.7±10.7%, 2nd and 3rd degree percentages were 2.11±4.64 and 0.62±5.2 respectively with 47 total inhalation injuries. Top burn etiologies were 244 (39.2%) scald and 175 (28.1%) flame with 249 (40%) coded etiology associated with food prep or consumption.

The majority of the burns occurred at home (93%). Time from injury to admission was 616.98±2199.42 minutes and time to first excision from admission was 4314.3 ± 5657.3 minutes. ICU and hospital length of stay were 12.7±18.3 and 8.73±13.3 days. In-hospital mortality was 31 (5%). Nineteen patients tested positive for COVID-19 during this time.

**Conclusions:**

Nearly half of all burn center admissions were for cooking related etiologies during this time. Time to admission was over 10 hours in a population dense area. More information of site specific pre-pandemic etiology and treatment data are needed to fully understand these initial findings. Further sub-analyses may also elucidate the influence of pandemic related behavioral changes as public health mandates evolved over time.

